# Spiral Sound Wave Transducer Based on the Longitudinal Vibration

**DOI:** 10.3390/s18113674

**Published:** 2018-10-29

**Authors:** Wei Lu, Yu Lan, Rongzhen Guo, Qicheng Zhang, Shichang Li, Tianfang Zhou

**Affiliations:** 1 Acoustic Science and Technology Laboratory, Harbin Engineering University of China, Harbin 150001, China; luwei@hrbeu.edu.cn (W.L.); guorongzhen@hrbeu.edu.cn (R.G.); Q.Zhang5@salford.ac.uk (Q.Z.); lsc1993@hrbeu.edu.cn (S.L.); 2 Key Laboratory of Marine Information Acquisition and Security (Harbin Engineering University), Ministry of Industry and Information Technology, Harbin 150001, China; 3 College of Underwater Acoustic Engineering, Harbin Engineering University of China, Harbin 150001, China

**Keywords:** spiral sound wave transducer, longitudinal vibration, orthometric dipole, finite element

## Abstract

A spiral sound wave transducer comprised of longitudinal vibrating elements has been proposed. This transducer was made from eight uniform radial distributed longitudinal vibrating elements, which could effectively generate low frequency underwater acoustic spiral waves. We discuss the production theory of spiral sound waves, which could be synthesized by two orthogonal acoustic dipoles with a phase difference of 90 degrees. The excitation voltage distribution of the transducer for emitting a spiral sound wave and the measurement method for the transducer is given. Three-dimensional finite element modeling (FEM)of the transducer was established for simulating the vibration modes and the acoustic characteristics of the transducers. Further, we fabricated a spiral sound wave transducer based on our design and simulations. It was found that the resonance frequency of the transducer was 10.8 kHz and that the transmitting voltage resonance was 140.5 dB. The underwater sound field measurements demonstrate that our designed transducer based on the longitudinal elements could successfully generate spiral sound waves.

## 1. Introduction

Spiral electromagnetic waves have been used in aircraft navigation for about 70 years [1]. Based on similar navigation principles, spiral sound waves have also shown potential applications in underwater navigation. In 2011, the Naval Undersea Warfare Center-Newport (NUWC–Newport, Newport, RI, USA) used only a single spiral sound wave beacon and one ITC-6050C hydrophone to achieve underwater navigation. The experimental navigation system is simpler, lighter and easier to use than the traditional underwater navigation system (e.g., a long baseline system, a short baseline system and an ultra-short baseline system). It shows many potentials for miniaturized underwater unmanned platform navigation. However, in order to improve the navigation distance and accuracy, low frequency and high power spiral sound wave transducers desperately require development.

Currently, the underwater acoustic transducers used to generating spiral sound waves are mainly achieved using two methods. One is the spiral sound wave transducer based on “physical spiral shape” proposed by Dzikowicz and Hefner in 2008 [2,3,4,5,6]. This transducer is mainly made of the 1–3 type piezoelectric composite materials, which were wrapped onto the surface of the spiral-shaped backup material so as to achieve the emission of spiral sound waves. The other transducer was proposed by Brown, in which the emission of spiral sound waves was realized by a radial polarized piezoelectric ring [7,8,9]. The electrode on the outer surface of the radial polarized piezoelectric ceramic ring was divided into four equal parts. Different excitation voltages were applied to four electrodes to excite the piezoelectric ring, resulting in orthogonal acoustic dipole fields with a phase difference of 90 degrees. However, the spiral sound wave transducer constructed with 1–3 piezoelectric composite materials may suffer from a low transmitting voltage response(TVR), large phase-horizontal azimuth fluctuation of generated waves, a low quality of the spiral sound field and a relatively high resonance frequency (e.g., 90 kHz). In addition, a spiral wave transducer designed with a radial polarized piezoelectric ceramic ring is limited by the size of the piezoelectric circular tube and the d31 piezoelectric constant. This makes the transducer incapable of working at low frequencies with high power efficiency [10,11].

In this paper, a cylindrical transducer based on eight longitudinal vibrating piezoelectric elements is proposed to emit spiral sound waves. In Section 2, we demonstrate the basic principles of the spiral wave generation ability of the transducer and analyze the excitation voltage distribution of the transducer for emitting spiral sound waves. Further, the electro-acoustic performance of the spiral acoustic transducer was simulated by the finite element (FE) method. The vibration characteristics and acoustic field characteristics of the transducer in water were examined in Section 3. In Section 4, a prototype of the spiral sound wave transducer is fabricated and measured according to the simulations and optimizations. The main findings are summarized in the Discussion.

## 2. The Generation of Spiral Sound Waves

### 2.1. Mechanism of Spiral Sound Wave Generation

**Figure 1:**
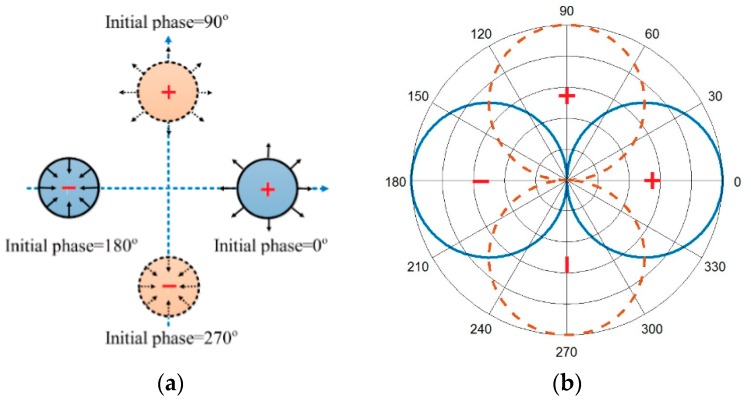
(**a**) The permutation of the four-point sound sources and their initial vibration phase in the *x*-*y* planes; (**b**) The directivity of the two pairs of sound sources.

We considered four point-sound sources with equal amplitude, and their phases were 0, 90, 180, and 270° respectively. One pair of point sources (blue) was aligned horizontally to form an acoustic dipole along the *x*-axis, and another pair (red) was aligned vertically to form an acoustic dipole along the *y*-axis. As a result, the system behaved as two pairs of acoustic dipoles, which were spatially orthogonal with the phase difference of 90°, as shown in Figure 1.

In spherical coordinates, the sound pressure of the two pairs of orthogonal acoustic dipoles with the phase difference of 90° was expressed as

(1) $$p_{1}\;\left(r,\theta ,\varphi \right)=P\cos\theta \cos\varphi e^{\left(-jkr\right)}e^{j\omega t}$$

(2) $$p_{2}\left(r,\theta ,\varphi \right)=P\cos\theta \sin\varphi e^{-jkr}e^{j\left(\omega t+\frac{\pi }{2}\right)}.$$

In addition, the superimposed sound pressure field was

(3) $$p\left(r,\theta ,\varphi ,t\right)=p_{1}+p_{2}=P\cos\theta \cos\varphi e^{j\left(\omega t-kr\right)}+P\cos\theta \sin\varphi e^{j\left(\omega t-kr+\frac{\pi }{2}\right)}.$$

Equation (3) can be simplified as

(4) $$p\left(r,\theta ,\varphi ,t\right)=P\cos\theta e^{j\left(\omega t-kr\right)}\left(\cos\varphi +j\;\sin\varphi \right)$$

according to Euler formula

(5) $$\;e^{j\varphi }=\cos\varphi +j\;\sin\varphi .$$

**Figure 2:**
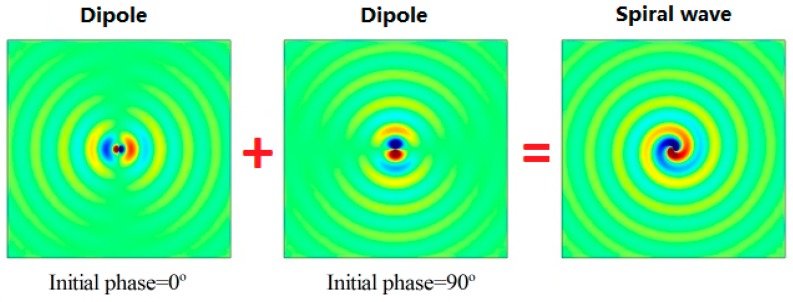
The spiral sound field generated by the superposition of two dipole sound fields.

Equation (4) can be expressed as

(6) $$p\left(r,\theta ,\varphi ,t\right)=P\cos\theta e^{j\left(\omega t-kr\right)}e^{j\varphi }$$

when θ = 0, Equation (6) reads as

(7) $$p\left(r,0,\varphi ,t\right)=Pe^{j\left(\omega t-kr\right)}e^{j\varphi }$$

where $\;e^{j\varphi }$ denotes the item of spiral line, namely, the spiral sound wave can be generated by two pairs of orthogonal dipole sound fields with a 90° phase difference on the horizontal plane. We assumed that the sound transmission was lossless and the simulation results of the spiral sound field generated by the superposition of two dipole sound fields are shown in Figure 2.

### 2.2. Excitation Mode of Spiral Sound Wave Transducer

**Figure 3:**
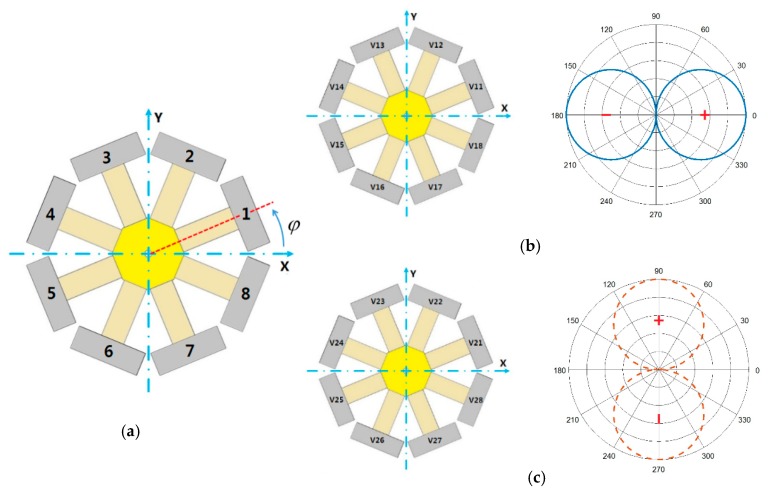
(**a**) The serial number of the LVPEs in the transducer; (**b**) the serial number of excitation voltages applied to the LVPEs for the dipole sound field along the *x*-axis and the direction of the sound field; (**c**) the serial number of excitation voltages applied to the LVPEs for the dipole sound field along the *y*-axis and the direction of the sound field.

To realize orthogonal acoustic dipoles, we used eight longitudinally vibrating piezoelectric elements (LVPEs) sharing one tail mass assembled by equal intervals along the circumferential direction and they were numbered 1–8 in a counterclockwise direction, as shown in Figure 3a [12,13,14,15]. The excitation voltage applied to each element satisfied the following equation in the circumferential direction:

(8) $$V\left(\varphi \right)=V_{0}\cos\varphi$$

where  φ  represents the angle between the center axis of the piezoelectric element and the *x*-axis. The transducer will behave as an acoustic dipole in the form of cosφ in the horizontal circumferential direction (*x*-axis direction) [16], as shown in Figure 3b. The excitation voltages applied to the LVPEs are listed in Table 1.

Similarly, if the excitation voltage took an acoustic dipole along the *y*-axis then the initial phase difference was 90°, as shown in Figure 3c. The excitation voltages, in this case, are listed in Table 2.

Therefore, to produce the orthogonal dipole sound fields with 90° of phase difference simultaneously, the two groups of excitaion voltages in Table 1 and Table 2 wereadded and applied to the LVPEs [17,18], as show in Table 3.

It can be seen in Table 3 that the amplitude of the excitation voltage applied to each element should be an equal value, while the initial phase was governed by an arithmetic progression with the tolerance of *π*/4.

## 3. Finite Element Simulation of the Spiral Sound Wave Transducer

### 3.1. The Structure and Size Design of Transducer

**Figure 4:**
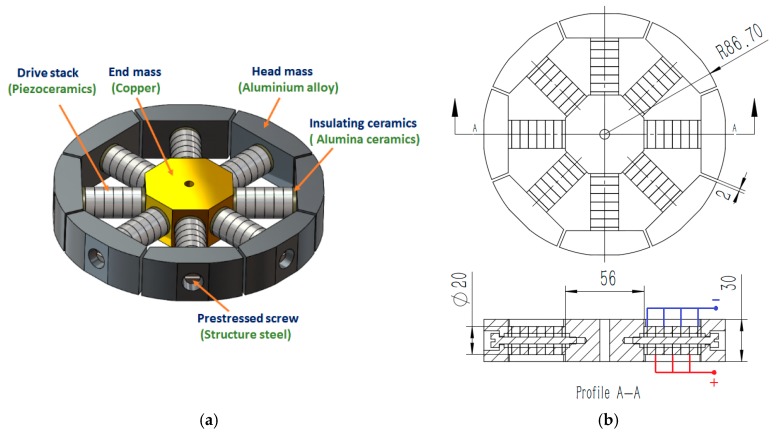
(**a**) The configuration of the spiral sound wave transducer; (**b**) the basic structure size and electrical connection of the transducer, the unit is mm.

Based on the principles demonstrated in Section 2, a transducer for transmitting spiral sound waves was designed and the FEM simulations were conducted. As shown in Figure 4, the transducer was composed of eight LVPEs, which were assembled equidistantly along the circumferential direction and connected to a communal central mass. The head mass of each element was manufactured to be arc-shaped to ensure the transducer adopted a cylindrical geometry. The angle of each head mass was 45°. The head masses were made from aluminum alloy (see Appendix A), the center mass was made from copper (see Appendix A), and the pre-stressed screws were made from structural steel (see Appendix A). The piezoelectric ceramic stack was assembled with a PZT-4 (see Appendix A) type hollow piezoelectric ceramic. Adjacent piezoelectric ceramics were polarized oppositely and excited in parallel. Since each LVPE worked under independent phase control, two insulating ceramic wafers, which were made from alumina ceramic (see Appendix A), were inserted at both ends. The basic structure and dimensions of the transducer are presented in Figure 4.

The dimensions of the transducer were 173.4 mm in diameter and 30 mm in height. The PZT-4 piezoelectric ceramics were manufactured with an outer diameter of 20 mm, an inner diameter of 10 mm, and a thickness of 6 mm, respectively. The pre-stressed screws were 5 mm in diameter. The arc-shaped aluminum alloy head masses were designed with a thickness of 20 mm and equal heights to that of the transducer.

### 3.2. The Finite Element Simulation of Transducer

**Figure 5:**
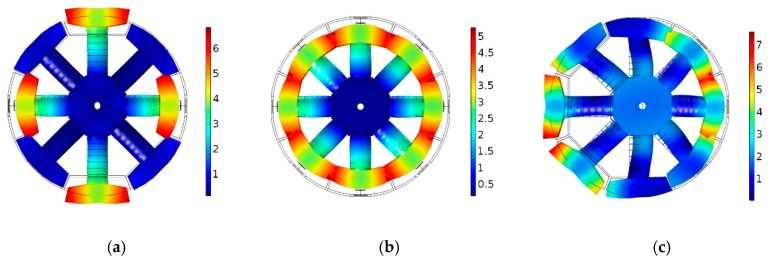
(**a**) The mode of vibration in quadrupole mode of the transducer; (**b**) the mode of vibration in monopole mode of the transducer; (**c**) the mode of vibration in dipole mode of the transducer. The calibrations of the color illustrations represent the relative values of the vibration displacement.

The commercial finite element software COMSOL was used to establish the finite element model of the transducer and the vibration modes of the transducer were obtained by the modal analysis [19,20,21]. The transducer based on the LVPEs had three basic modes for transmitting spiral sound waves. The first one was a quadrupole mode at 8.52 kHz, the second one was a monopole mode at 8.78 kHz and the third one was a dipole mode at 11.28 kHz, as shown in Figure 5.

**Figure 6:**
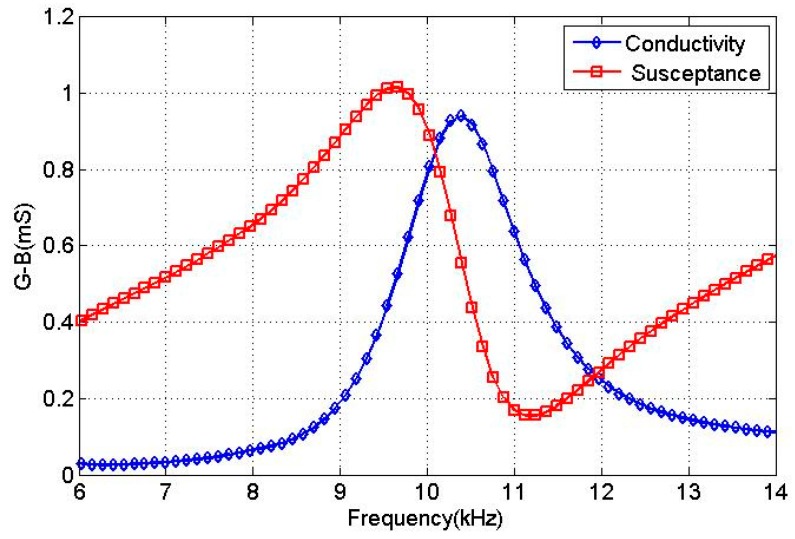
The conductivity curves of the transducer in water.

Since the spiral sound wave was synthesized by the dipole sound field, the resonant frequency of the transducer in air was determined as 11.28 kHz. Meanwhile, a finite element model of the transducer in water was built. The excitation voltages were given by Table 3. The electro-acoustic characteristics of the transducer in water were obtained by harmonic response analysis. The calculated conductivity curves for the transducer in water are given in Figure 6.

**Figure 7:**
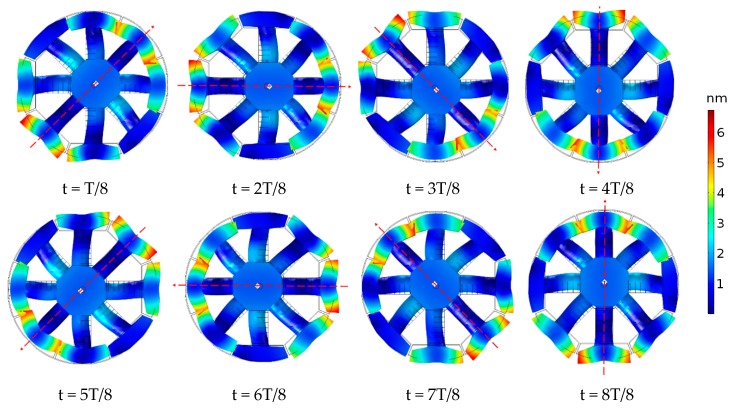
The change for the positive direction to dipole vibration of the transducer in one excitation period. The excitation voltage was 1 V.

From Figure 6, the conductance curve of the transducer only shows one peak at 10.5 kHz within the frequency range 6–15 kHz and the conductance was 0.9 mS. The excitation voltages in Table 3 correspond to the dipole mode resonance of the transducer. The vibration shapes of the transducer over one excitation period, T, at the resonant frequency are illustrated in Figure 7. An evolution of the dipole vibration mode can be clearly observed, which corresponds to the transmitting of the spiral sound.

The red arrows in Figure 7 denote the positive direction of the dipole vibration. One can note that the dipole rotates in the clockwise direction by *π*/4 during a T/8 period and that means that the maximum sound pressure of the dipole rotates in a circle over one excitation period, which agrees with the definition of the spiral sound wave in Equation (7).

**Figure 8:**
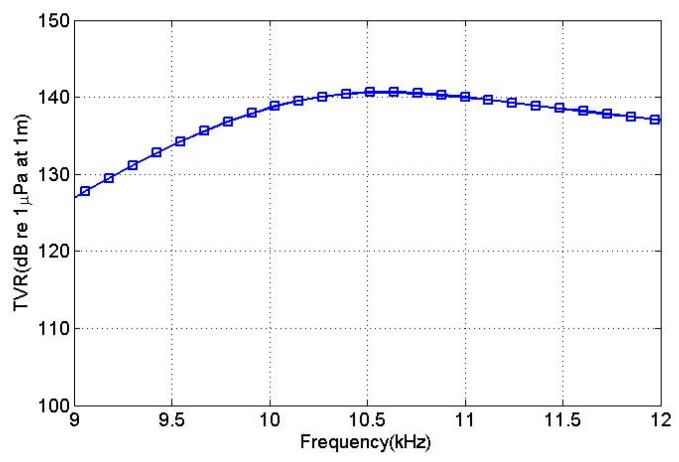
The transmitting voltage response of the transducer in water.

Figure 8 depicts the simulated transmitting voltage response (TVR) of the transducer in water. The maximum TVR of the transducer in the water was 140.5 dB.

**Figure 9:**
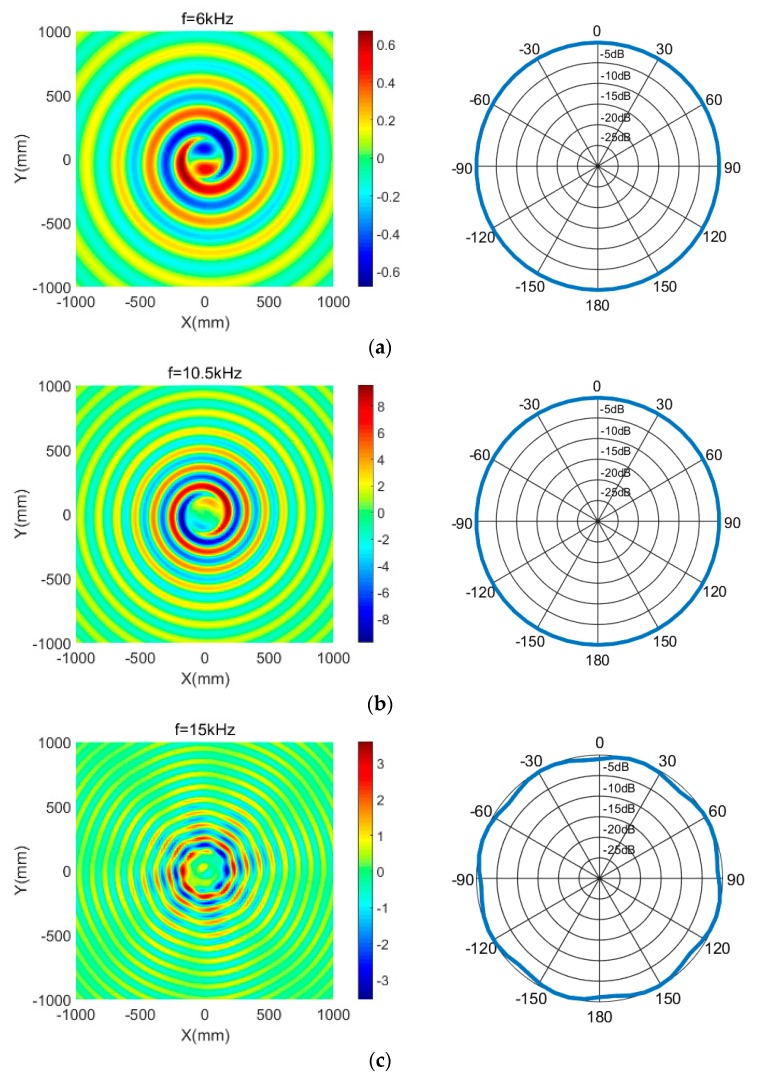
(**a**) The sound pressure distribution and sound pressure level directivity of the transducer at 6 kHz; (**b**) the sound pressure distribution and sound pressure level directivity of the transducer at 10.5 kHz; (**c**) the sound pressure distribution and sound pressure level directivity of the transducer at 15 kHz.

Figure 9 shows the horizontal pressure fields and the pressure level directionality of the transducer under 1 V excitation, at working frequencies of 6, 10.5 and 15 kHz, respectively. We found that the transducer produced spiral sound fields over the frequency range 6–15 kHz and it featured horizontal non-directionality, except for the frequency of 15 kHz. Further, it was found that the horizontal directionality fluctuated by 1.5 dB due to the reduction of the directionality opening angle of a single LVPE.

**Figure 10:**
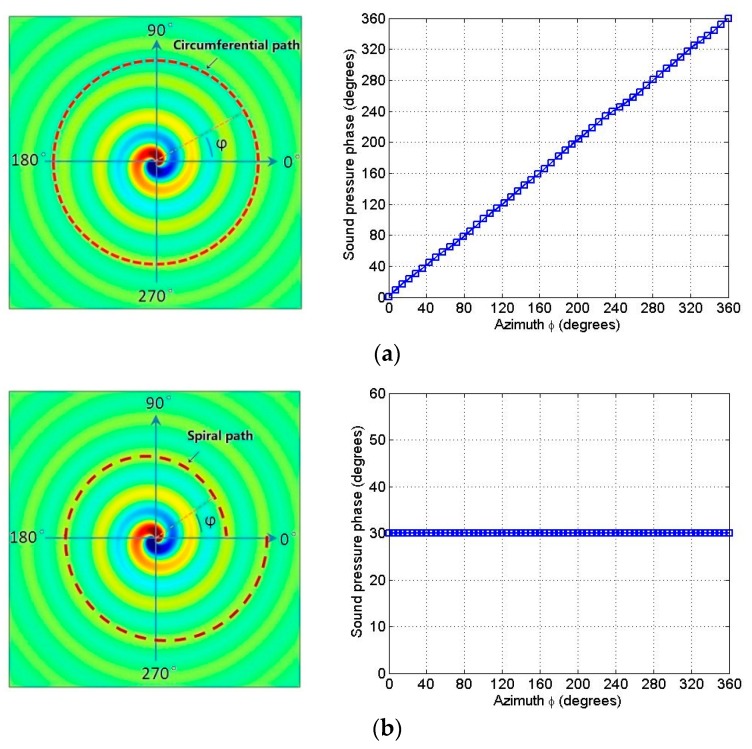
(**a**) The emulation phase angle of the spiral wave as a function of the physical azimuthal angle *ϕ* along the circular path at the resonance frequency 10.5 kHz; (**b**) the emulation phase angle of the spiral wave as a constant of the physical azimuthal angle *ϕ* along the spiral line path at the resonance frequency 10.5 kHz.

The relationship between the phase of the sound pressure and the azimuth angle *ϕ* was analyzed in the circumferential path at the far field of the spiral sound wave transducer at the resonant frequency of 10.5 kHz. From Figure 10a, it is clear that the sound pressure phase on the circular path was proportional to the azimuth angle. In the spiral path, the phase of the sound pressure did not change with respect to the azimuth angle *ϕ* and remained constant, as shown in Figure 10b. It should be noted that the curves shown in Figure 10a,b have no ups and downs, which indicates that the sound field produced by the transducer is of high quality.

## 4. Fabrication and In-Water Testing of Transducer Prototype

**Figure 11:**
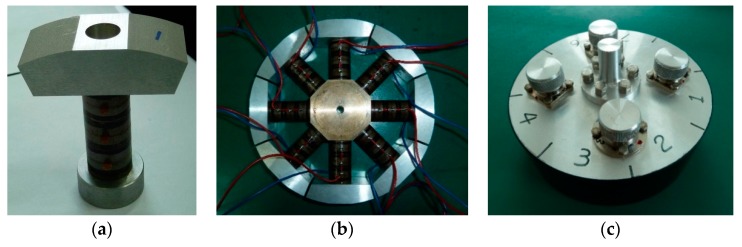
(**a**) A single LVPE; (**b**) the internal structure of the transducer prototype. All parts of the transducer were bonded with epoxy resin; (**c**) the transducer prototype after watertight encapsulation with polyurethane.

Figure 11 is a picture of the real transducer prototype, in which Figure 11a is the single LVPE, Figure 11b is the internal structure of the transducer prototype and Figure 11c is the transducer after watertight encapsulation with polyurethane. The final size of the transducer prototype was184.3 mm in diameter, 57 mm in height, and a weight of 4 kg in air.

### 4.1. The Measurement of Spiral Sound Field

The spiral sound wave transducer was placed at the origin of a Cartesian coordinate system and two hydrophones were placed at *x*1(*r*_0_,0,*ϕ*_1_) and *x*2(*r*_0_,0,*ϕ*_2_), respectively. The horizontal angle difference between them was

(9) $$\varphi_{2}-\varphi_{1}=\Delta \varphi_{1}.$$

The sound pressure signals measured by two hydrophones were

(10) $$s_{1}=M_{e}Pe^{j\left(\omega t-kr_{0}\right)}e^{j\mu \varphi_{0}}$$

(11) $$s_{2}=M_{e}Pe^{j\left(\omega t-kr_{0}\right)}e^{j\mu (\varphi_{0}+\Delta \varphi )}$$

where the initial phases of two signals at *t* = 0 were

(12) $$Arg_{1}=-kr_{0}+\varphi_{0}$$

(13) $$Arg_{2}=-kr_{0}+(\varphi_{0}+\Delta \varphi ).$$

The phase difference between the output signals of the two hydrophones was

(14) $$Arg_{2}-Arg_{1}=\Delta \varphi .$$

**Figure 12:**
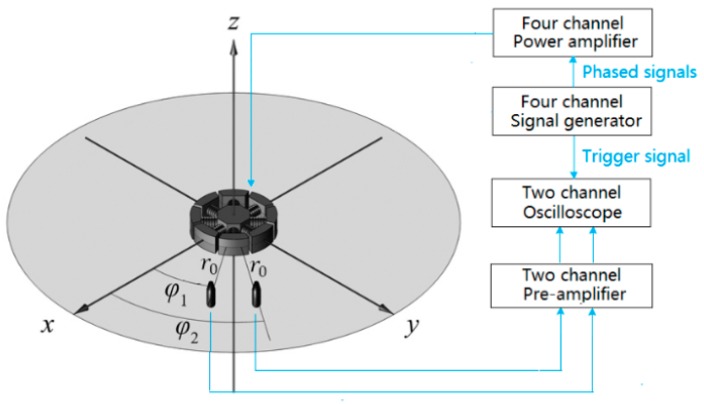
Test transducer placement and equipment connection.

If the phase difference between the output signals of the two hydrophones, ∆𝜑, obtained during the test was equal to ∆$\varphi_{1}$, Figure 11a was obtained and the sound field could be considered as a spiral sound field. The placement of the transducer prototype for the test and the connection diagram of the test equipment are shown in Figure 12. The horizontal angle difference between the two hydrophones was 15 degrees and the $r_{0}$ was 2 m away from the sound center.

### 4.2. The Test of Spiral Acoustic Wave Transducer

**Figure 13:**
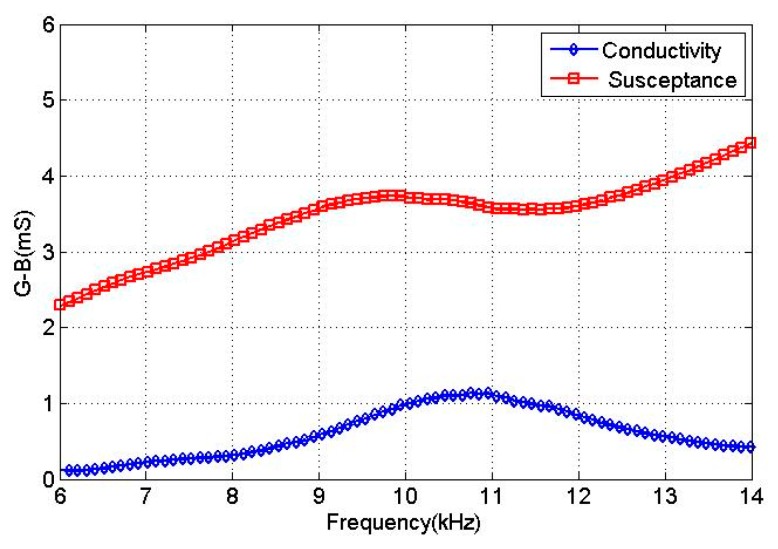
Conductance curves of the transducer under dipole vibration in water.

First, a WK6500 impedance analyzer was used to test the conductance curve of the transducer in the dipole vibration mode in water and then determine the resonance frequency of the spiral sound wave transducer. As shown in Figure 13, the transducer made a resonant frequency in the dipole vibration mode in water at 10.8 kHz and its conductance value was 1.2 mS. In the experiment, we found that, when transmitting spiral sound waves, the resonant frequency of the transducer was 10.8 kHz, which differed by 0.3 kHz from the simulation result.

**Figure 14:**
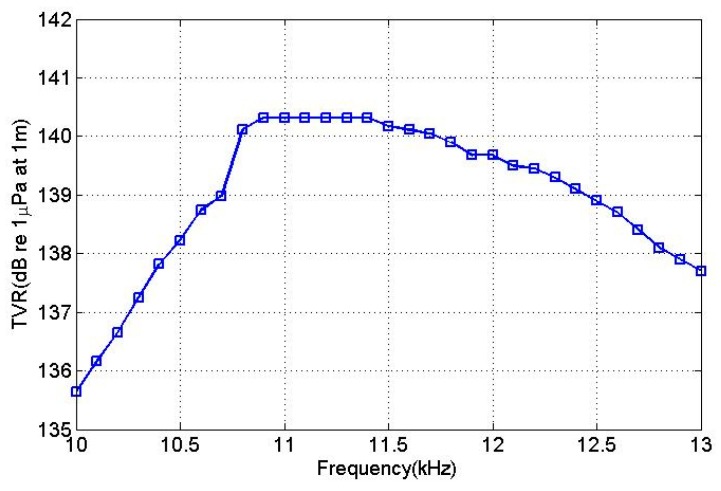
Transducer emission voltage response curve.

The driving voltage was applied according to the excitation voltage distributions given in Table 3 and the transmitting voltage response curves of the transducer were obtained, as shown in Figure 14. The maximum transmitting voltage response value at the resonance frequency was 140.5 dB, which differed by 0.5 dB from the simulation result.

**Figure 15:**
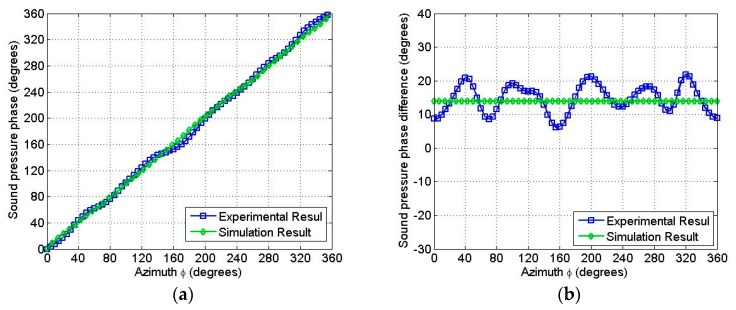
(**a**) The received signal sound pressure phase obtained by one hydrophone measurement, where the transducer rotated 360 degrees at 10.8 kHz; (**b**) The received sound pressure phase difference from two hydrophones by rotating the transducer for 360 degrees at 10.8 kHz.

The phase characteristics of the sound field transmitted by the transducer were obtained by secondary calculation from the measured data. From Figure 15a, it is evident that the azimuth *ϕ* was proportional to the sound pressure phase from the hydrophone and matched the simulation results. In Figure 15b, it is observed that the sound pressure phase difference from two hydrophones had a 15-degree azimuth difference. Furthermore, it can be seen that there was a fluctuation of 10 degrees at the phase of the spiral sound wave in the circumferential direction and the average phase difference had a 5% difference from the azimuth angle difference of the hydrophones. The phase characteristics of the sound field conform to a spiral sound wave, thus, it can be concluded that the spiral sound wave could be transmitted by the transducer based on the LVPEs.

## 5. Conclusions

In this paper, eight longitudinal vibration piezoelectric elements were used to form a cylindrical transducer for transmitting spiral sound waves and the electro-acoustic performance of the transducer was simulated and analyzed using the finite element method. The transducer had a resonance frequency of 10.8 kHz and the transmitting voltage response of 140.5 dB by acoustic measurement. The sound phase on the circular path in the far-field transmitted by the transducer was proportional to the azimuth angle, which followed the characteristics of the spiral sound wave. So we conclude that the transducer based on longitudinally vibrating piezoelectric elements was capable of transmitting spiral sound waves.

Compared to the physical spiral transducer and the split piezoelectric ring type spiral sound wave transducer, our transducer, based on longitudinal vibration piezoelectric elements, had the following advantages:

(1) The transducer was composed of longitudinal vibration piezoelectric elements, which used a d33 piezoelectric coefficient, thus, improving the sound power radiation of the transducer.
(2) The design and manufacture of the longitudinal vibration elements were not limited by the size of the piezoelectric ceramic material, which enabled the transducer to have a lower resonance frequency and thus improved the working distance of the underwater acoustic equipment that used spiral sound waves to achieve positioning and navigation.
(3) The longitudinal vibration piezoelectric elements feature simple manufacturing processes and are of low cost, which made it possible to select longitudinal vibration piezoelectric elements with a consistent performance. These properties ensure the quality of the spiral sound field emitted by the transducer.

## Figures and Tables

**Table 1:** The voltages distribution of the dipole sound field excited along the *x*-axis for the longitudinally vibrating piezoelectric elements (LVPEs).

Voltage Number	Voltage Value	Normalization	Voltage Number	Voltage Value	Normalization
*V*11	$V_{0}\cos\left(22.5^{{}^{\circ}}\right)$	1	*V*15	$V_{0}\cos\left(202.5^{{}^{\circ}}\right)$	−1
*V*12	$V_{0}\cos\left(67.5^{{}^{\circ}}\right)$	0.4	*V*16	$V_{0}\cos\left(247.5^{{}^{\circ}}\right)$	−0.4
*V*13	$V_{0}\cos\left(112.5^{{}^{\circ}}\right)$	−0.4	*V*17	$V_{0}\cos\left(292.5^{{}^{\circ}}\right)$	0.4
*V*14	$V_{0}\cos\left(157.5^{{}^{\circ}}\right)$	−1	*V*18	$V_{0}\cos\left(337.5^{{}^{\circ}}\right)$	1

**Table 2:** The voltage distribution of the dipole sound field excited along the *y*-axis for the LVPEs.

Voltage Number	Voltage Value	Normalization	Voltage Number	Voltage Value	Normalization
*V*21	$V_{0}\sin\left(22.5^{{}^{\circ}}\right)e^{j\frac{\pi }{2}}$	0.4*j*	*V*25	$V_{0}\sin\left(202.5^{{}^{\circ}}\right)e^{j\frac{\pi }{2}}$	−0.4*j*
*V*22	$V_{0}\sin\left(67.5^{{}^{\circ}}\right)e^{j\frac{\pi }{2}}$	*j*	*V*26	$V_{0}\sin\left(247.5^{{}^{\circ}}\right)\;e^{j\frac{\pi }{2}}$	−*j*
*V*23	$V_{0}\sin\left(112.5^{{}^{\circ}}\right)e^{j\frac{\pi }{2}}$	*j*	*V*27	$V_{0}\sin\left(292.5^{{}^{\circ}}\right)\;e^{j\frac{\pi }{2}}$	−*j*
*V*24	$V_{0}\sin\left(157.5^{{}^{\circ}}\right)e^{j\frac{\pi }{2}}$	0.4*j*	*V*28	$V_{0}\sin\left(337.5^{{}^{\circ}}\right)\;e^{j\frac{\pi }{2}}$	−0.4*j*

**Table 3:** The voltage distribution of LVPEs for transmitting spiral sound waves using a two dipole sound field.

Voltage Number	Voltage Value	Normalization	Voltage Number	Voltage Value	Normalization
*V*31	*V*11 *+ V*21 *=* 1 + 0.4*j*	$e^{j\frac{2\pi }{8}}$	*V*35	*V*15 *+ V*25 *= −*1 *−* 0.4*j*	$e^{j\frac{10\pi }{8}}$
*V*32	*V*12 *+ V*22 = 0.4 + *j*	$e^{j\frac{4\pi }{8}}$	*V*36	*V*16 *+ V*26 *= −*0.4 *− j*	$e^{j\frac{12\pi }{8}}$
*V*33	*V*13 *+ V*23 *= −*0.4 + *j*	$e^{j\frac{6\pi }{8}}$	*V*37	*V*17 *+ V*27 *=* 0.4 *− j*	$e^{j\frac{14\pi }{8}}$
*V*34	*V*14 *+ V*24 *= −*1 + 0.4*j*	$e^{j\frac{8\pi }{8}}$	*V*38	*V*18 *+ V*28 *=* 1 *−* 0.4*j*	$e^{j\frac{16\pi }{8}}$

## Appendix A

Material properties

1. Aluminum alloy:
  Density: 2790 kg/m^3^, Young’s modulus: 71.5 GPa, Poisson’s ratio: 0.34.
2. Copper:
  Density: 8960 kg/m^3^, Young’s modulus: 110 GPa, Poisson’s ratio: 0.35.
3. Structure steel:
  Density: 7850 kg/m^3^, Young’s modulus: 200 GPa, Poisson’s ratio: 0.30.
4. Alumina ceramic:
  Density: 3800 kg/m^3^, Young’s modulus: 350 GPa, Poisson’s ratio: 0.22.
5. Piezoelectric ceramic (PZT-4) [22]:

**Table A1:** Material properties of piezoelectric ceramic (PZT-4).

C_11_^E^ (10^10^ N/m^2^)	C_12_^E^ (10^10^ N/m^2^)	C_13_^E^ (10^10^ N/m^2^)	C_33_^E^ (10^10^ N/m^2^)	C_44_^E^ (10^10^ N/m^2^)	C_66_^E^ (10^10^ N/m^2^)
13.9	7.78	7.43	11.5	2.56	3.06
**Density (kg/m^3^)**	$\varepsilon_{11}^{s}/\varepsilon_{0}$	$\varepsilon_{33}^{s}/\varepsilon_{0}$	$e_{31}$ **(C/m^2^)**	$e_{33}$ **(C/m^2^)**	$e_{15}$ **(C/m^2^)**
7500	730	635	−5.2	15.1	12.7
